# Primary desmoplastic small round cell tumor of the duodenum

**DOI:** 10.1186/2047-783X-19-38

**Published:** 2014-07-06

**Authors:** Qi Liu, Nan Liu, DeXing Chen

**Affiliations:** 1 General Surgery Department, Qian Wei Hospital of Jilin province, Changchun City, Jilin Province, China

**Keywords:** chemotherapy, desmoplastic small round cell tumor, duodenum, surgery

## Abstract

The desmoplastic small round cell tumor (DSRCT) is an extremely rare tumor that mainly affects adolescents and mostly involves the abdominal and pelvic peritoneum. A 14-year-old girl presented with intermittent epigastric pain; abdominal computed tomography and upper gastrointestinal barium X-ray revealed an 8 cm × 10 cm space-occupying mass in the duodenal region. The patient underwent pancreaticoduodenectomy and the final pathologic diagnosis was DSRCT. Although multi-agent systemic chemotherapy was given, the patient died of metastasis 8 months later. Early diagnosis and surgical treatment with adjuvant chemotherapy seems to be the best treatment choice for this disease.

## Background

The desmoplastic small round cell tumor (DSRCT) is a rare, aggressive, and malignant tumor, first described by Gerald in 1989 [1]. It mainly affects adolescents and mostly involves the abdominal or pelvic peritoneum. Diagnosis is usually based on the tumor’s histological and immunohistochemical features. Despite multimodality treatments, DSRCT has a poor prognosis. To the best of our knowledge, only one case of duodenal DSRCT has been reported in the English-language literature [2], making this case the second case to be reported in the duodenal region so far.

## Case presentation

**Figure 1:**
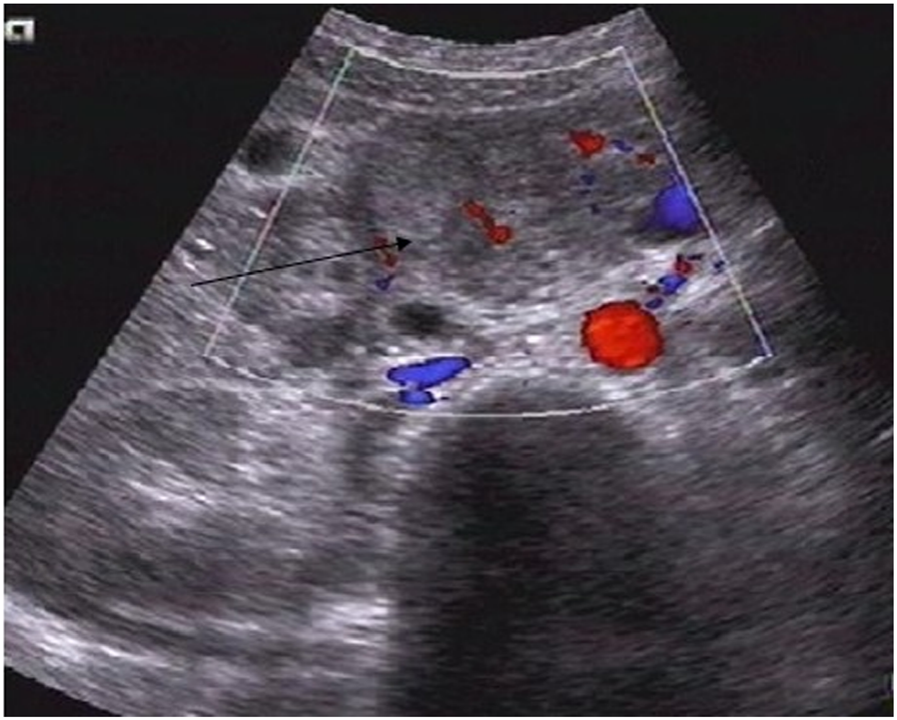
Sonography revealed a 7.1 cm × 7.2 cm hypoechoic mass at the wall of the duodenum and pancreas (arrow).

**Figure 2:**
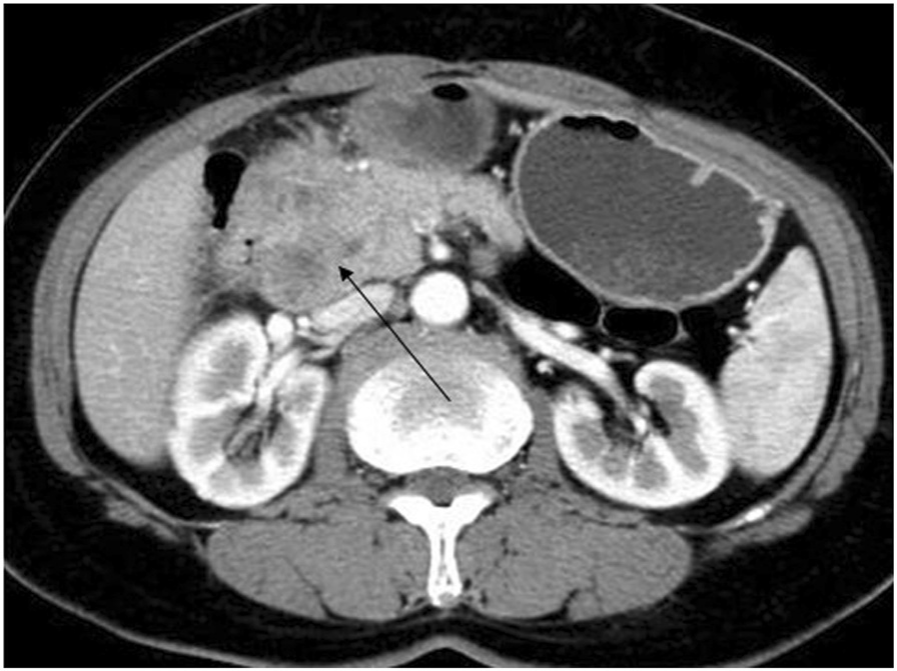
Abdominal computed tomography (CT) showed an 8 cm × 10 cm lobulated solitary mass encircling the duodenum with low attenuation and inhomogeneous enhancement (arrow).

**Figure 3:**
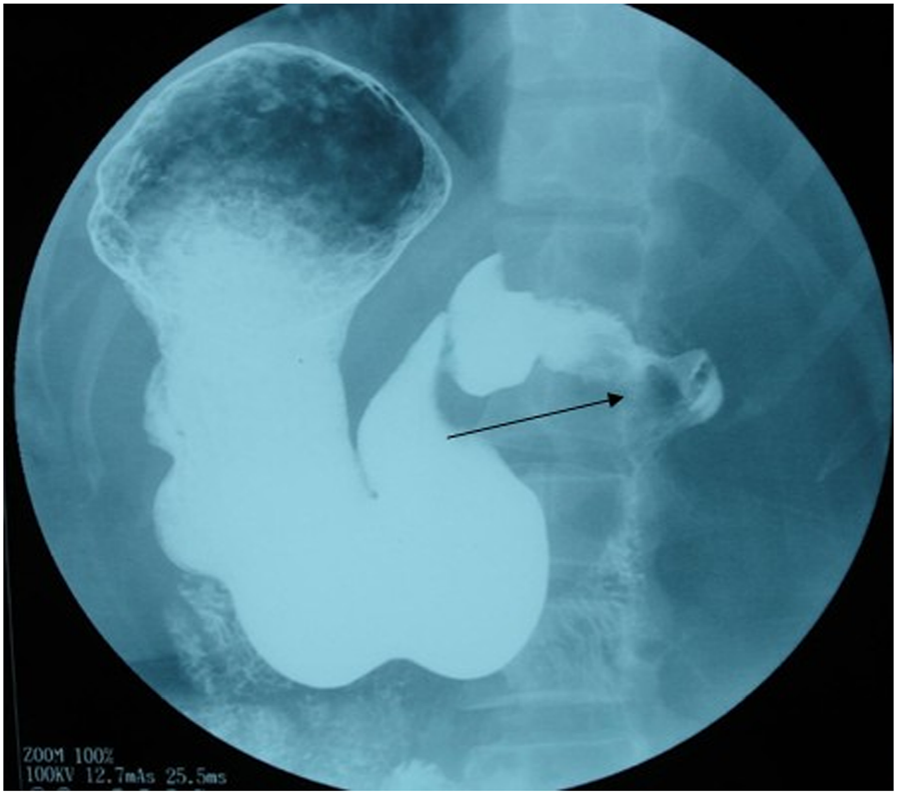
Upper gastrointestinal barium X-ray revealed a defect in the duodenum with a tendency to displace the duodenum (arrow).

On 13 February 2008, a 14-year-old girl presented with a history of intermittent epigastric pain and bloody vomit for one month. There was no history of fever, weight loss, or jaundice. Further examination showed tenderness in the otherwise soft upper quadrant of the abdomen, without palpable masses. The patient was diagnosed with chronic cholecystitis and treated with antibiotics for a week without relief. Hemoglobin concentration and red blood cell count were 55 g/l, 2.92 × 10^12^ /l, respectively, while the concentration of carbohydrate antigen 19-9 was within normal range. Abdominal ultrasonography revealed a 7.1 cm × 7.2 cm hypoechoic mass around the duodenum and pancreas (Figure 1). Computed tomography (CT) also detected an 8 cm × 10 cm lobulated solitary mass encircling the duodenum, with low attenuation and inhomogeneous enhancement (Figure 2). There was no evidence of metastasis to local or distant organs. An upper gastrointestinal barium X-ray revealed a defect in the duodenum and a space-occupying mass, invading retroperitoneally with a tendency to displace the duodenum (Figure 3). The patient refused duodenoscopy and was diagnosed as having a duodenal tumor before surgery. On surgery, we found a solid and irregular mass about 10 cm × 8 cm × 8 cm in the wall of duodenum without complete capsule. A frozen biopsy was performed and revealed malignant tumor features similar to malignant lymphoma or undifferentiated malignant tumor, but failed to identify the specific type or origin of the tumor. The tumor originated from the descending part of duodenum, and then invaded the pancreatic caput, gallbladder, and multiple para-aortic lymph nodes. A pancreaticoduodenectomy was performed to remove part of the duodenum, the gallbladder, and adjacent lymph nodes. The patient was discharged from hospital 10 days later with an uneventful recovery.

**Figure 4:**
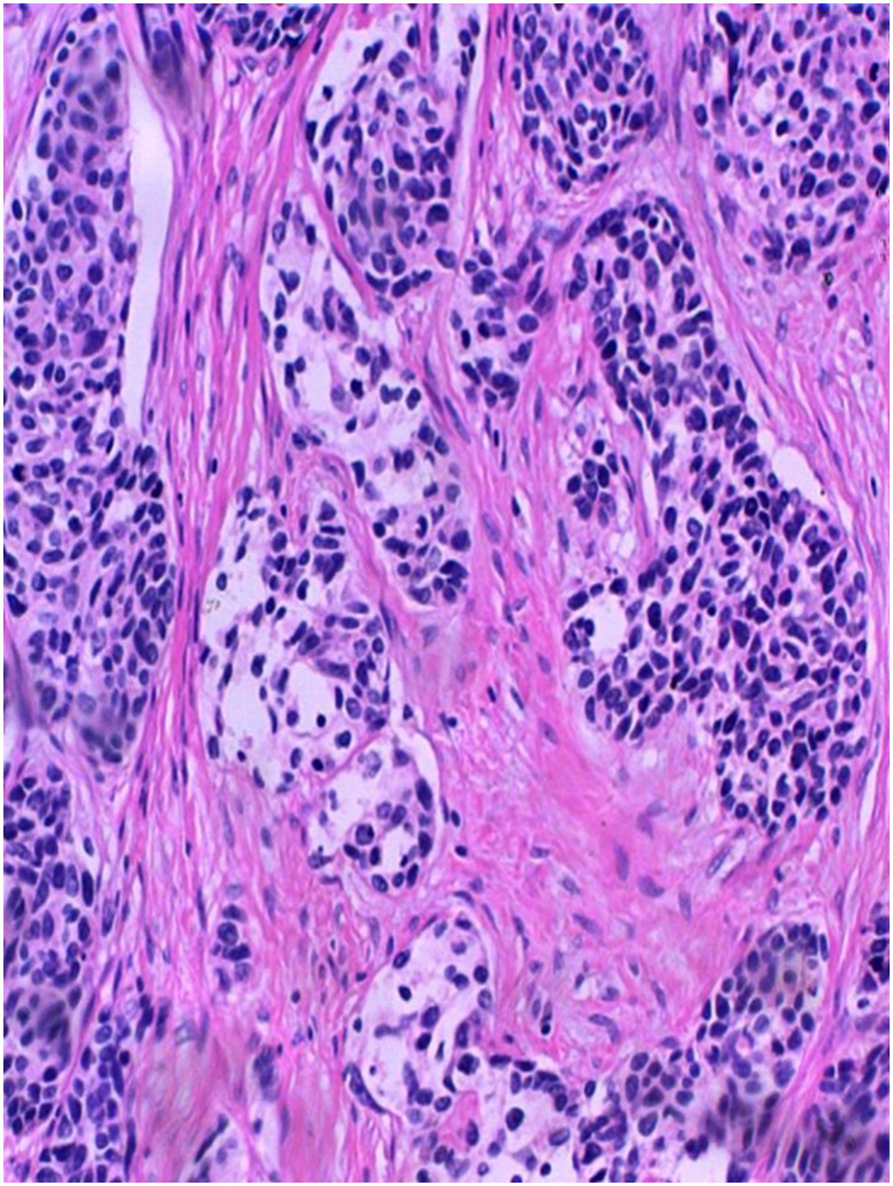
**Immunohistochemistry reveals nests of hyperchromatic round and focally spindle-shaped tumor cells in a desmoplastic background.** Some cells are arranged in well-defined nests, which are delimited by a cellular desmoplastic stroma (H & E, 200×).

**Figure 5:**
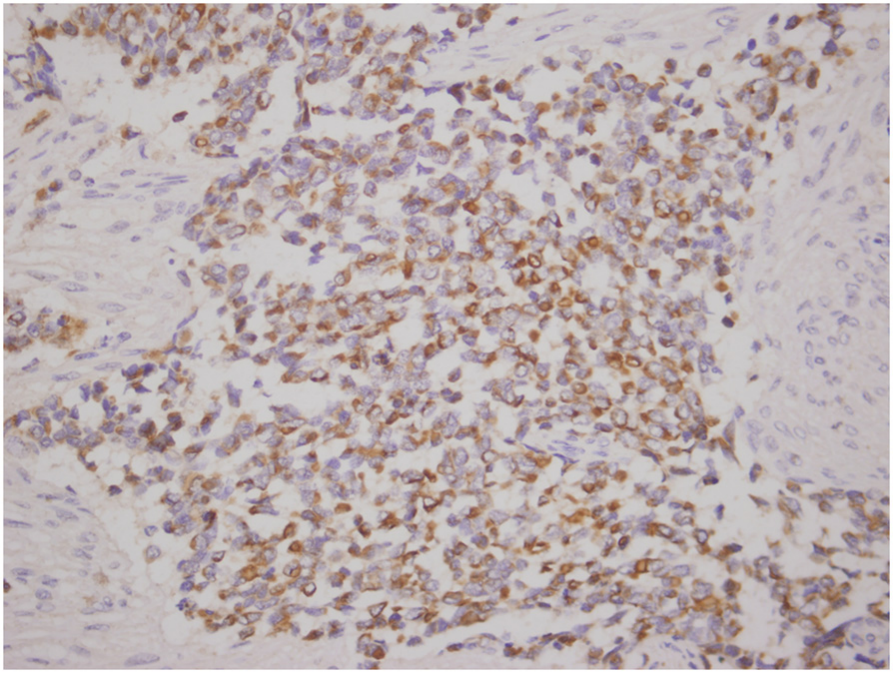
**Tumor cells stain positive for neuron-specific enolase.** (200×).

**Figure 6:**
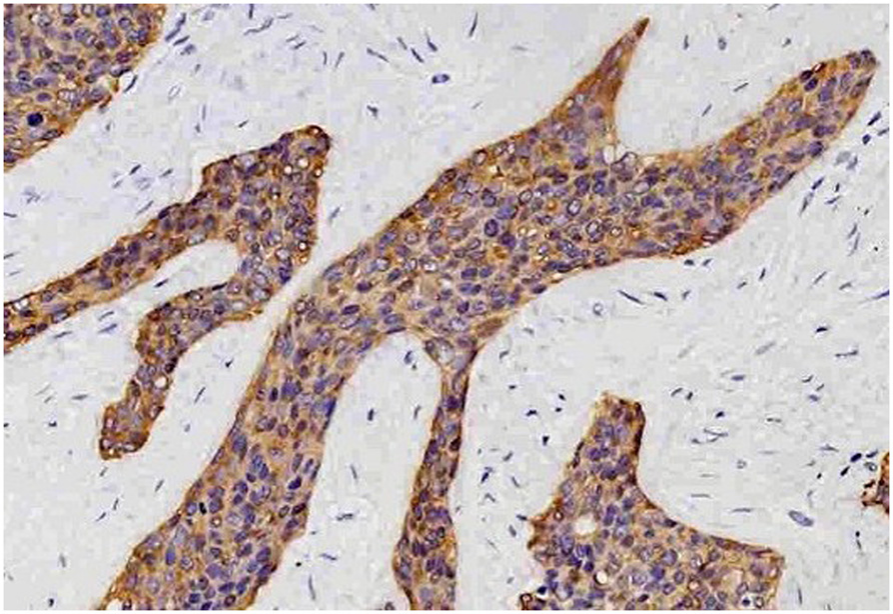
**Tumor cells stain positive for keratin.** (200×).

Postoperative examination of the specimen showed a tumor 8 cm × 10 cm in diameter. The tumor consisted of nests of ‘small blue cells’ with scant cytoplasm embedded in a densely fibrotic stroma and focal tubule formation. There were also nests of hyperchromatic round and focally spindle-shaped tumor cells in a desmoplastic background. Some cells were arranged in well-defined nests, which were delimited by a cellular desmoplastic stroma (Figure 4). Immunohistochemical findings showed that the tumor cells were diffusively positive for vimentin and CD99 antigen. Focal reactivity to neuron-specific enolase and keratin were observed (Figures 5,6), while tests for carcinoembryonic antigen, epithelial membrane antigen, and cytokeratin proved negative. Histological and immunohistochemical findings confirmed the diagnosis of DSRCT.

Eight weeks later, the patient was readmitted with nausea and vomiting. Abdominal color Doppler flow imaging showed a 7.4 cm × 6.7 cm irregular-echo mass with rich a blood supply. The patient was then treated with two-stage multi-agent chemotherapy. The longest diameter of the tumor decreased by 10% after two cycles of carboplatin and duoxitasai. Continued chemotherapy was performed every three weeks in two cycles after the patient’s release from hospital. However, the tumor was not sensitive to the chemotherapy and the patient’s condition deteriorated progressively; she died of multi-organ metastasis 6 months later.

## Discussion

The DSRCT is a rare and highly malignant tumor. Its growth is multinodular, involving serosal surfaces, such as the peritoneum, pleura, tunica vaginalis, and scrotum. Morphologically, DSRCTs are characterized by the formation of nests of small round cells proliferating in a cellular fibrous stroma. The DSRCT belongs to the family of ‘small round blue cell tumors’; nevertheless, molecular biology has proved that DSRCT is a unique tumor, which is different from other types of small round cell tumor. The genetic characterization of DSRCT is a chromosomal translocation of t(11; 22) (p13;q12) between the Ewing’s sarcoma (EWS) gene on chromosome 22 and the Wilms’ tumor (WT1) gene on chromosome 11, leading to a EWS-WT1 fusion transcript. The characteristic translocation t(11;22) (p13;q12) is specific for DSRCTs. This fusion product causes a loss of the tumor suppressor function of WT1 and a putative upregulation of various families of growth factors from the EWS gene [3]. The differential diagnosis includes other small round cell tumors, such as metastatic neuroblastoma, Ewing’s sarcoma, small cell carcinoma, and lymphoma, rhabdoid tumor, and small cell osteosarcoma. The presence of perinuclear dot-like immunostaining with desmin strongly suggests DSRCT [4].

Typical cases of DSRCT in the intra-abdominal cavity or gastrointestinal tract are accompanied by abdominal mass or pain, similar to other gastrointestinal tumors. The most useful radiographic method is CT with intravenous contrast. Imaging typically reveals multiple low-attenuation peritoneal soft tissue with a regular contour. Most masses are located within the mesentery, omentum, and paracolic gutter or along abdominopelvic peritoneal surfaces. Sometimes the lymph nodes and retroperitoneum can be involved but this is not typical in DSRCT. Ascites and solitary to multiple metastases nodules can also present in some patients. Tumors without an apparent primary organ-based distribution can be very suspicious for DSRCT. Currently, the prognosis of DSRCT is still poor because of its highly aggressive and progressive malignant character, together with multifocal presentation. The optimal treatment remains to be determined.

Total surgical excision is recommended only for non-metastatic DSRCTs. However, complete resection is usually impossible at an advanced stage [5], and postoperative systemic adjuvant chemotherapy should be initiated as soon as possible. Aggressive surgical resection combined with multi-agent adjuvant chemotherapy can relieve symptoms and improve the outcome of advanced DSRCTs. Unfortunately, most chemotherapy agents are only temporarily effective [6]. Although Farhat *et al.* recommended cisplatin, etoposide, cyclophosphamide, doxorubicin, or epirubicin as first-line alternative drugs, no consistent responses to chemotherapy are reported [7].

Despite aggressive treatment, the survival rate remains disappointing. According to a literature review and our experience, we recommend surgical debulking followed by aggressive chemotherapy for DSRCT. The correlation between intense chemotherapy combined with gross total resection and prolonged lifespan is high. Radiotherapy is only recommended for salvage therapy, but not for normal treatment, since the isolation may have less impact and effectiveness. Moreover, side effects may outweigh treatment. For these advanced-stage patients, symptomatic treatment is more practical as it alleviates acute discomfort; however, it still leads to a poor outcome. Aggressive multimodality therapy with immunotherapy or bone marrow ablation and dose-intensive chemotherapy with autologous peripheral blood stem cell support may have potential benefit for DSRCTs and may be a promising new approach [8]. Recently, continuous hyperthermic peritoneal perfusion at the time of complete tumor resection has been developed as an adjunct to treat DSRCTs; this may prolong disease-free survival in selected cases [9].

## Conclusions

We described a second case of DSRCT at the duodenal region. After tumor surgical ablation, the patient received multi-agent systemic chemotherapy, but she died of multiple metastases some months later. The dismal prognosis of DSRCT at the duodenal region is similar to that of DSRCT at the abdominopelvic region. According to a literature review and our experience, surgical debulking followed by aggressive chemotherapy is still the best treatment for DSRCT, mainly in non-metastatic tumors. However, the optimal treatment remains to be determined for this aggressive tumor.

## Consent

Written informed consent was obtained from the patient’s next of kin for publication of this case report and any accompanying images. A copy of the written consent is available for review by the editor-in-chief of this journal.

## Abbreviations

CT: computed tomography; DSRCT: desmoplastic small round cell tumor; EWS: Ewing’s sarcoma; H & E: hematoxylin and eosin; WT1: Wilms’ tumor.

## Competing interests

The authors declare that they have no competing interests.

## Authors’ contributions

QL conceived and wrote the manuscript; NL revised it critically for important intellectual content; DXC gave final approval to the version to be published. All authors read and approved the final manuscript.

## References

[B1] Gerald WL Rosai J Desmoplastic small cell tumor with divergent differentiation Pediatr Pathol 1989 9 177 183 10.3109/15513818909022347 2473463

[B2] Kim JW Park JH Cho HJ Kwon JH Koh Y Kim SJ Kim SH Lee SH Im SA Kim YT Kim WH A case of desmoplastic small round cell diagnosed in a young female patient Cancer Res Treat 2009 41 4 233 236 10.4143/crt.2009.41.4.233 20057970 PMC2802845

[B3] Roberts P Burchill SA Beddow RA Wheeldon J Cullinane C Lewis IJ A combined cytogenetic and molecular approach to diagnosis in a case of desmoplastic small round cell tumor with a complex translocation (11;22;21) Cancer Genet Cytogenet 1999 108 1 19 25 10.1016/S0165-4608(98)00103-4 9973919

[B4] Ordonez NG Desmoplastic small round cell tumor: I. a histopathologic study of 39 cases with emphasis on unusual histological patterns Am J Surg Pathol 1998 22 1303 1313 10.1097/00000478-199811000-00001 9808123

[B5] Talarico F Iusco D Negri L Belinelli D Combined resection and multi-agent adjuvant chemotherapy for intra-abdominal desmoplastic small round cell tumour: case report and review of the literature G Chir 2007 28 367 370 17915050

[B6] Lippe P Berardi R Cappelletti C Massacesi C Mattioli R Latini L Cellerino R Desmoplastic small round cell tumour: a description of two cases and review of the literature Oncology 2003 64 14 17 10.1159/000066514 12457026

[B7] Farhat F Culine S Lhommé C Duvillard P Soulié P Michel G Terrier-Lacombe MJ Théodore C Schreinerova M Droz JP Desmoplastic small round cell tumors: results of a four-drug chemotherapy regimen in five adult patients Cancer 1996 77 1363 1366 10.1002/(SICI)1097-0142(19960401)77:7<1363::AID-CNCR21>3.0.CO;2-Z 8608516

[B8] Bisogno G Ferrari A Rosolen A Scarzello G Garaventa A Arcamone G Carli M Sequential intensified chemotherapy with stem cell rescue for children and adolescents with desmoplastic small round-cell tumor Bone Marrow Transplant 2010 45 5 907 911 10.1038/bmt.2009.248 19802018

[B9] Hayes-Jordan A Green H Fitzgerald N Xiao L Anderson P Novel treatment for desmoplastic small round cell tumor: hyperthermic intraperitoneal perfusion J Pediatr Surg 2010 5 5 1000 1006 20438942 10.1016/j.jpedsurg.2010.02.034

